# Processes for Implementing Community Health Worker Workforce Development Initiatives

**DOI:** 10.3389/fpubh.2021.659017

**Published:** 2021-06-24

**Authors:** Colleen Barbero, Theresa Mason, Carl Rush, Meredith Sugarman, Aunima R. Bhuiya, Erika B. Fulmer, Jill Feldstein, Naomi Cottoms, Ashley Wennerstrom

**Affiliations:** ^1^Centers for Disease Control and Prevention, Atlanta, GA, United States; ^2^Community Resources, LLC, San Antonio, TX, United States; ^3^Center for Healthcare Value and Equity, Louisiana State University Health Sciences Center, New Orleans, LA, United States; ^4^Institute of Health Policy, Management and Evaluation, University of Toronto, Toronto, ON, Canada; ^5^Penn Center for Community Health Workers, University of Pennsylvania Health System, Philadelphia, PA, United States; ^6^Tri-County Rural Health Network, Helena-West Helena, AR, United States; ^7^Department of Behavioral and Community Health Sciences, School of Public Health, Louisiana State University Health Sciences Center, New Orleans, LA, United States

**Keywords:** community health worker, workforce development, promotora, promotor, community health representative

## Abstract

**Introduction:** The objective of this observational, cross-sectional study was to identify, document, and assess the progress made to date in implementing various processes involved in statewide community health worker (CHW) workforce development initiatives.

**Methods:** From September 2017 to December 2020, we developed and applied a conceptual model of processes involved in implementing statewide CHW initiatives. One or more outputs were identified for each model process and assessed across the 50 states, D.C., and Puerto Rico using peer-reviewed and gray literature available as of September 2020.

**Results:** Twelve statewide CHW workforce development processes were identified, and 21 outputs were assessed. We found an average of eight processes implemented per state, with seven states implementing all 12 processes. As of September 2020, 45 states had a multi-stakeholder CHW coalition and 31 states had a statewide CHW organization. In 20 states CHWs were included in Medicaid Managed Care Organizations or Health Plans. We found routine monitoring of statewide CHW employment in six states.

**Discussion:** Stakeholders have advanced statewide CHW workforce development initiatives using the processes reflected in our conceptual model. Our results could help to inform future CHW initiative design, measurement, monitoring, and evaluation efforts, especially at the state level.

## Introduction

A community health worker (CHW) is a frontline public health worker who is a trusted member of and/or has a close understanding of the community served (1). Community health workers, including *promotor(a)s* and community health representatives (CHRs), build relationships and trust with people experiencing health inequities based on shared life experiences. CHWs provide tailored support based on understanding people's experiences, needs, and preferences. Research has shown that interventions engaging CHWs have led to positive health, social, and economic outcomes for individuals (2–5) and communities (6).

The COVID-19 pandemic has presented many opportunities and challenges for the CHW workforce and their employers. Early in the pandemic, the National Association of Community Health Workers (NACHW) found that many CHWs were laid off or experienced reduced work hours or activities (7). However, many stakeholders, including federal and state public health agencies, healthcare payers, and private healthcare companies, have bolstered support for employment of this critical workforce during the pandemic (8, 9). This new interest in CHW employment, combined with ongoing challenges, such as sustainable financing for CHW positions and scaling their integration into health delivery systems, make CHW workforce development a salient contemporary public health issue (10–13).

Statewide CHW workforce development initiatives can include state level strategies and activities focused on enhancing capacity of CHWs and current or potential CHW employers. According to the National Academy for State Health Policy (NASHP), nearly every state reported activity to support the CHW workforce in 2017 (14). As of June 2016, nearly half of states including D.C. had enacted laws pertaining to the CHW workforce (15). Over the last decade, federal agencies, including the Centers for Disease Control and Prevention (CDC) and Centers for Medicare and Medicaid Services (CMS), have provided funding that state public health agencies and their partners have leveraged to implement statewide CHW workforce development initiatives (16, 17).

Process theory provides a useful framework for analyzing the implementation of complex interventions (18–20) and can be applied to statewide CHW workforce development initiatives. The objective of this observational, cross-sectional study was to identify, document, and assess the progress made to date in implementing the processes involved in statewide CHW workforce development initiatives. Results could help to inform future CHW initiative design, measurement, monitoring, and evaluation efforts, especially at the state level (21, 22).

## Methods

From September 2017 to December 2020, researchers at the CDC partnered with experts in CHW workforce development and related policies to: (1) engage stakeholders to develop and test a conceptual model of the processes involved in implementing statewide CHW workforce development initiatives and (2) apply the model to assess CHW workforce development initiative processes and outputs across the 50 states, D.C., and Puerto Rico. The Tulane University Social and Behavioral Institutional Review Board determined this project to be exempt.

### Conceptual Model Development

Development of the conceptual model began with review of relevant literature and models (21, 22). Findings from this review were used to draft an initial model that was reviewed by nine stakeholders, including CHWs, healthcare and community employers, state public health agency staff, and CHW workforce training experts, during a virtual meeting in February 2018. The stakeholders were recruited through our professional networks and represented several regions of the U.S. Criteria for stakeholder selection included (1) a history of leading CHW workforce development initiatives and programs related to training, certification, and/or sustainable financing, and (2) being a CHW or having worked directly with CHWs. In this meeting we took notes as stakeholders suggested edits to the model, ordered the processes in a logical manner (although it was acknowledged that these initiatives are often not linear in practice), and discussed potential outputs.

### Assessment of Model Processes for Three States

**Figure 1:**
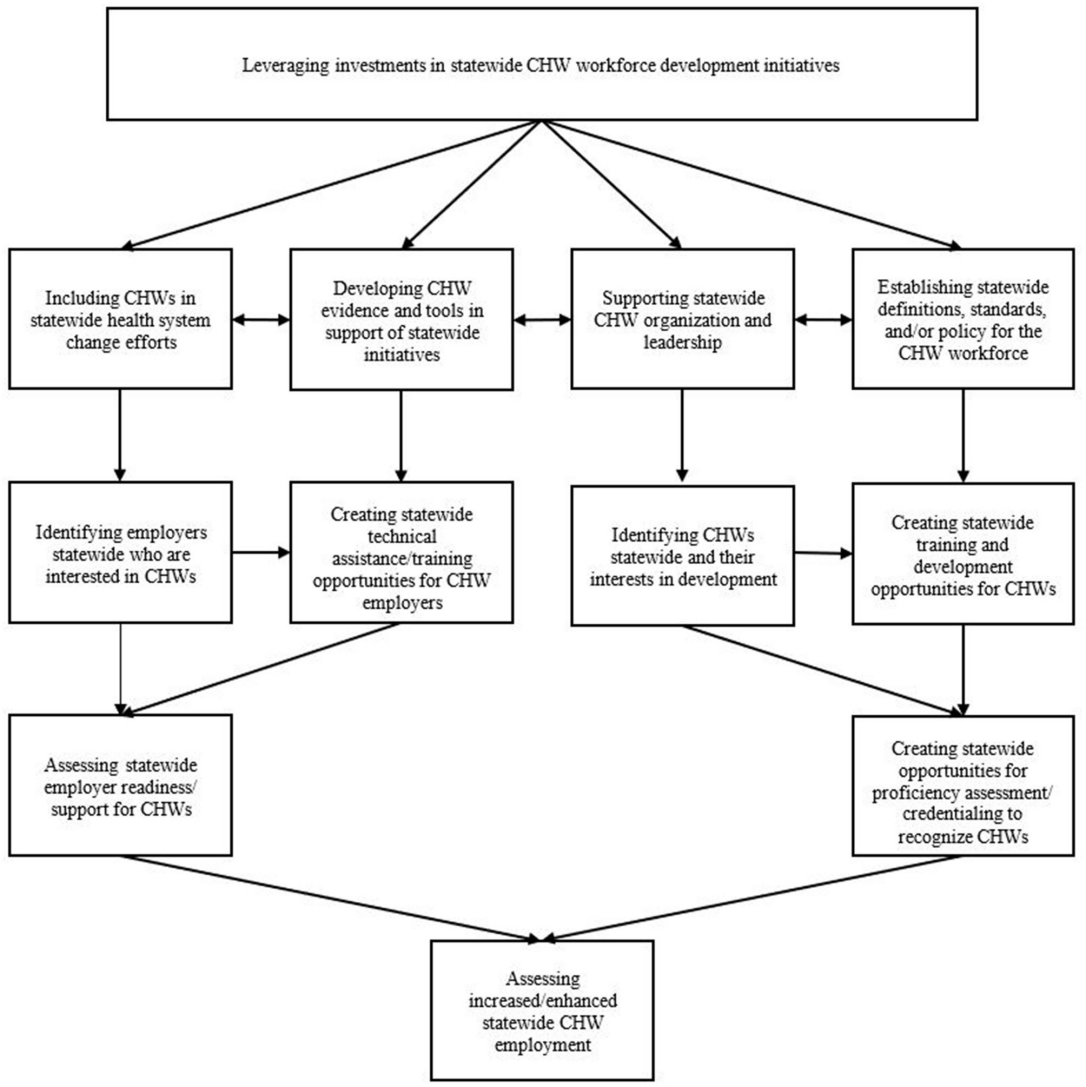
Conceptual model of processes involved in statewide community health worker (CHW) workforce development initiative implementation.

After the stakeholder meeting, we finalized the model (Figure 1) and selected three states for initial application. State selection criteria included: evidence of significant historical and current CHW activity; current or prior workforce development efforts led by the state public health agency and/or statewide CHW organization; and documentation of progress in studying the CHW workforce (e.g., strategic planning for workforce expansion and assuring appropriate CHW selection and training). In June 2018, we conducted group interviews with three key informants in each of three states (*n* = 9). Interviews included CHW initiative leaders from state health department, CHWs who were currently serving in a leadership role in a statewide CHW organization, and CHW employers. To encourage participants to speak openly, we promised that identifying information, including participants' state, would not be included in publications or presentations. One researcher from our team led the group interview in each state, using the conceptual model as a guide for the discussion, while a second team member took extensive notes. Afterwards, participants provided additional resources (e.g., state meeting minutes, grant applications, and training reports) to offer more information. We used the group interview notes and other sources to develop a technical report (unpublished) detailing the statewide CHW workforce development processes implemented in each of the three states.

### Identification of Outputs and Assessment for 50 States, D.C., and Puerto Rico

After assessing the model processes present in three states based on interviews and extant documents, we developed a systematic peer-reviewed and gray literature collection and assessment procedure for application to all 50 states, D.C., and Puerto Rico (“states”). From May 2018 to September 2020, we conducted searches for literature for each state using: internet databases and search engines (PubMed, Google Scholar, and Google); references and citations from existing literature; and relevant funder (CDC, CMS, state health department, academic and research institution, and training and workforce development organization) and third sector (CHW organization and coalition) websites. State-specific search strings were created using the terminology from the boxes in the conceptual model. For example, one search string was: “[state name]” AND “community health worker” AND “training”. National CHW resources (14–17) were also searched for relevance to individual states.

Next, we documented statewide CHW workforce development processes and outputs for each state. We compared findings from state documentation with the notes from our virtual stakeholder meeting and the technical report to develop a list of 21 process outputs, with at least one output identified for each model process (Table 1). Then we re-reviewed all sources to ensure that all 21 outputs were assessed for each state. Data quality was ensured by having at least two researchers independently review all the documents, processes, and outputs for each state and the research team review the aggregate findings. The full list of sources reviewed and the final assessment of outputs for each state are included in the Supplementary File.

**Table 1:** Community health worker (CHW) workforce development initiative processes and outputs for the 50 states, D.C. and Puerto Rico as of September 2020^a^.

**Conceptual model process**	**# of states out of 52**	**Output(s) associated with this process**	**# of states out of 52**
Leveraging investments in statewide CHW workforce development initiatives	48	Centers for Medicare and Medicaid Services State Innovation Model w/1 or more process	27
		Centers for Disease Control and Prevention 1305 or 1422 Program w/1 or more process	33
Including CHWs in statewide health system change efforts	46	Medicaid Managed Care Organizations or Health Plans include CHWs	20
		Patient-Centered Medical Homes/Health Homes include CHWs	11
		Medicaid 1115 Waiver includes CHWs	10
		Medicaid State Plan Amendment includes CHWs	10
		Community Health/Care Teams include CHWs	6
		Accountable Care Organizations include CHWs	4
		Accountable Communities of Health include CHWs	4
Supporting statewide CHW organization and leadership	41	Statewide CHW organization comprised mostly of CHWs	31
Establishing statewide definitions, standards, and/or policy for CHW workforce	47	Statewide multi-stakeholder CHW coalition or other entity focused on advancing the CHW workforce	45
		Stakeholders have adopted a statewide CHW definition	34
		Stakeholders have adopted statewide CHW core competencies or scope of practice	33
Developing evidence and tools in support of statewide CHW initiatives	42	State-level report on CHW workforce development	35
Identifying CHWs statewide and their interests in development	35	Statewide survey of the CHW workforce	26
Identifying employers statewide who are interested in CHWs	33	Statewide survey of CHW employers	22
Creating statewide training/technical assistance opportunities for CHW employers	37	Statewide training program for CHW employers	15
Creating statewide training and development opportunities for CHWs	46	Statewide CHW training program(s)/apprenticeship available	36
Creating statewide opportunities for proficiency assessment/credentialing to recognize CHWs	36	Statewide CHW certification process available (does not include certificate programs)	18
Assessing statewide employer readiness/support for CHWs	10	Conducting routine, statewide monitoring of CHW employer readiness	5
Assessing increased/enhanced statewide CHW employment	13	Conducting routine, statewide monitoring of CHW employment	6
Minimum, maximum # of processes addressed by a state	2, 12		
Average # of processes addressed by a state	8		

a *See Supplementary File for state-specific results*.

## Results

### Conceptual Model

Our final conceptual model includes 12 logically ordered processes that can be involved in implementation of statewide CHW workforce development initiatives (Figure 1). The first process in the model involves stakeholders leveraging financial and other investments for the development and implementation of statewide initiatives, and the last process in the model involves efforts to assess increased and enhanced CHW employment statewide. The right side of the model includes processes focused on CHWs and the left side includes processes focused on employers.

### Processes and Outputs Across States

As of September 2020, most states had implemented most of the processes from our model, with an average of eight out of 12 processes implemented per state, and seven states implementing all 12 processes (Table 1). Nine states had implemented less than half of the 12 processes, with a minimum of two processes implemented. Results of our assessment of the 21 process outputs across states are provided in the Table 1.

### Outputs by Process

1. *Leveraging investments for statewide CHW workforce development initiatives*: We found that as of September 2020, nearly every state (48 states) had leveraged a financial investment for CHW workforce development initiative implementation. For example, CMS and CDC funding were leveraged during 2013–2018 to address one or more of our model processes in 27 and 33 states respectively (16, 17).
2. *Including CHWs in statewide health system change efforts:* Most states (46 states) were also implementing this process, but since not every state chose the same approach, there were different outputs. The most common outputs across states were CHW inclusion in: State Innovation Models (27 states); Medicaid Managed Care Organizations and Health Plans (20 states); Patient-Centered Medical Homes or Health Homes (11 states); Medicaid Waivers (10 states); and Medicaid State Plan Amendments (10 states).
3. *Developing evidence and tools in support of statewide CHW initiatives*: Slightly fewer states (42 states) were working to develop an evidence base and tools in support of statewide CHW initiatives. For example, stakeholders in Minnesota developed a CHW employer toolkit (23), and the Pathways Community HUB model, which includes training for CHWs and data collection, has been implemented in 20 states so far (24). In 35 states, stakeholders have published a report about CHW workforce development in their state.
4. *Establishing statewide definitions, standards, and/or policy for CHW workforce:* Stakeholders in 47 states have been working to develop statewide infrastructure to support the CHW workforce, with a multi-stakeholder CHW coalition present in most of these states (45 states). Two common outputs of this process were a statewide CHW definition, often based on the American Public Health Association definition (1), and recognition of core competencies, often based on the national CHW Core Consensus (C3) Project (25), in 34 and 33 states respectively.
5. *Supporting statewide CHW organization and leadership*: In 41 states, stakeholders were supporting CHW workforce organization and leadership, with statewide CHW organizations formed in 31 states; in Nebraska, Utah, and Wisconsin, this included a CHW section of the state public health association.
6. *Identifying CHWs statewide and their interests in development:* In 35 states, stakeholders had made efforts to engage CHWs across the state to learn about their work and interests. A little over half of states (26 states) had conducted at least one statewide survey of CHWs.
7. *Creating statewide training and development opportunities for CHWs:* In 46 states, stakeholders were working on creating training opportunities for CHWs. In 36 states, a CHW training program had been made available to CHWs statewide (Alaska, Iowa, Pennsylvania, and Wisconsin offered CHW apprenticeships).
8. *Identifying employers statewide who are interested in CHWs:* Compared with efforts to identify CHWs, fewer states (33 states) were working to identify CHW employers across the state. Statewide CHW employer surveys were also less common (in 22 states); in most of the states with employer surveys (20 states), CHWs were also surveyed.
9. *Creating statewide training/technical assistance opportunities for CHW employers:* We found that stakeholders were providing technical assistance and training about CHWs to employers in 37 states. However, statewide training programs about CHWs for employers were also less common (in 15 states). Most of these trainings were created for CHW supervisors.
10. *Creating statewide opportunities for proficiency assessment/credentialing to recognize CHWs:* Stakeholders in 36 states were undertaking efforts to advance professional recognition for CHWs. These efforts included establishing CHW certification, offering certified CHW titling, and/or granting CHW certificates. As of September 2020, 18 of these states had made a statewide certification process available to CHWs.
11. *Assessing statewide employer readiness/support for CHWs:* Far fewer states (10 states) had efforts to assess employers across the state on their readiness for employing CHWs. Only five of these states had systems in place for routine monitoring of employer readiness. As one example, in Michigan, the statewide CHW alliance conducts biannual employer surveys, which gather information on employer support for the statewide CHW training program (26).
12. *Assessing increased/enhanced statewide CHW employment:* Similarly, only 13 states had efforts to advance assessment of statewide CHW employment, with only six states having routine monitoring systems, often supported by the statewide CHW organization or coalition.

## Discussion

This study describes the processes achieved in implementing statewide CHW workforce development initiatives as of September 2020. Findings are relevant to state level planning and evaluation frameworks (21, 22, 27). We found that states have largely implemented CHW workforce development initiatives using the processes reflected in our conceptual model. Repeated assessments using our model and outputs could provide important information to track improvements and gaps in practice.

Although we found that 47 states have made efforts to establish statewide definitions, standards, and policy for the CHW workforce, it is important to note that fewer states (41 states) had efforts dedicated to organizing the CHW workforce. Similarly, while 45 states had a multi-stakeholder CHW coalition in September 2020, 31 states had a statewide CHW organization comprised primarily of CHW members (Table 1). These two types of entities often have different purposes, with the CHW organization(s) typically serving as the “voice” for the CHW workforce in the state. The opportunity to partner with a statewide CHW organization may have a wide range of benefits, including enabling the successful execution of workforce studies and full participation of CHWs in the formation of policy (28). CHW organizations can also help to lead decision making about whether or not to pursue CHW certification or another form of professional recognition.

Our discovery that training programs and surveys were less common for CHW employers than they were for CHWs is also important because employer understanding of and appreciation for the distinctive CHW role and core attributes of CHW candidates are vital for implementation of successful CHW programs (29). Regional approaches could support wider availability of employer training across states (30, 31), but nuances in local culture, availability of community resources, and local and state regulations that may affect the CHW workforce can also be considered. Furthermore, the impact of the statewide CHW employer technical assistance and training that we found in the 37 states will also be important to assess. However, as of September 2020, we found only a handful of states with systems in place to monitor statewide changes in employer readiness and CHW employment (Table 1). Repeated administrations of existing surveys were one way to advance statewide CHW workforce monitoring and evaluation. For example, surveys conducted in Michigan and Minnesota have been able to track improvements in CHW employment rates and job benefits, such as sick and personal leave, health insurance, mileage reimbursement, and vacation accrual (23, 26).

Securing sustainable financing for CHWs remains a key objective among stakeholders. We found the inclusion of CHWs in several different Medicaid financing mechanisms, with the most common being Managed Care Organizations (MCOs) and Health Plans (in 20 states) (Table 1). While this reflects progress, more examples and opportunities may exist; for example, in 2017, 39 states had at least one Medicaid MCO (32). Despite being widely promoted as a pathway to sustainability, we found the presence of a Medicaid State Plan Amendment or Section 1115 Waiver that explicitly included CHWs both in only about one-fifth of states (10 states each).

There are some limitations to this study. The assessment relied on publicly available information, which may become quickly outdated and fail to identify all applicable outputs. It is likely that we captured only the major, documented, centralized efforts, and in the future, the field would benefit from collecting more data on the many local and community level efforts that are contributing to the advancement of this versatile, diverse public health workforce. Another limitation is that some of the efforts we included in this assessment may not have been sustained, as comprehensive financing for statewide CHW workforce development initiatives remains an ongoing challenge.

Furthermore, we were not able to assess statewide CHW employment numbers as an output, due to many challenges in using available data, including the use of CHW definitions that overlap with definitions for other health care professionals. For this reason, we did not count reporting to the Bureau of Labor Statistics CHW occupational category (10) as routine monitoring of CHW employment. Additionally, we are aware that some CHW workforce members may not perceive themselves to be CHWs, and some community-based clinical health professionals may mistakenly identify as CHWs (33). This issue will need to be addressed if CHW counts are to be used for monitoring and evaluation.

While our study was able to assess the presence of a statewide CHW organization in each state, another next step for research could be to assess CHW organization co-leadership in statewide initiatives (34). Lastly, as data collection improves, it may be possible to estimate the impact of statewide CHW workforce development approaches on population health outcomes and health equity. While we found that statewide CHW certification is a common approach for workforce development implemented among states (Table 1), it remains only one option for advancing the professional recognition of CHWs. It will be crucial to assess for any unintended consequences of this policy on the CHW workforce. For example, depending on how it is designed, statewide CHW certification could pose a barrier to practice. Researchers might also consider how statewide CHW certification compares with alternative approaches chosen by stakeholders, such as increased support for CHW training, efforts to educate employers about CHW roles, and/or certifying employer or training programs instead of CHWs.

Overall, this article illustrates how CHW workforce development has been advanced across states. Many opportunities still exist to support statewide CHW organizations, scale statewide financing mechanisms, and improve employment data collection. Additional support for CHW workforce development could help to increase the engagement, reach, and impact of this critical workforce.

## Data Availability Statement

The original contributions presented in the study are included in the article/Supplementary Material, further inquiries can be directed to the corresponding author/s.

## Author Contributions

CB, TM, CR, MS, AB, EF, NC, JF, and AW contributed to developing the conceptual model, conceptualization of the manuscript, and contributed to reviewing and editing the manuscript. CR and TM conducted the group interviews. MS and AW developed the technical report. CB, TM, CR, MS, AB, EF, and AW completed the state coding. All authors contributed to the article and approved the submitted version.

## Conflict of Interest

CR and TM were employed by the company Community Resources, LLC. The remaining authors declare that the research was conducted in the absence of any commercial or financial relationships that could be construed as a potential conflict of interest.
